# Supplementary material for: From sequence to enzyme mechanism using multi-label machine learning
Source: BMC Bioinformatics. 2014 May 19;15:150. doi: 10.1186/1471-2105-15-150 (PMC4229970; doi:10.1186/1471-2105-15-150)
Supplement: Additional file 2 — Java code of ml2db. Additional file ml2db_code.tar.gz contains the Java source code to run the multi-label machine learning experiments and save the results to database. The code’s Javadoc is included. [file 1471-2105-15-150-S2.zip › additional file 2/ml2db/ml2db/doc/test/ml2db/al/utility/neighbourhood/NearestNeighboursByFreqUtilityTest.html]

NearestNeighboursByFreqUtilityTest


JavaScript is disabled on your browser.


- Overview
- Package
- Class
- Use
- Tree
- Deprecated
- Index
- Help

- Prev Class
- Next Class

- Frames
- No Frames

- All Classes

- Summary:
- Nested |
- Field |
- Constr |
- Method

- Detail:
- Field |
- Constr |
- Method


test.ml2db.al.utility.neighbourhood

## Class NearestNeighboursByFreqUtilityTest

- java.lang.Object
- - test.ml2db.al.utility.neighbourhood.NearestNeighboursByFreqUtilityTest

- ---

    

  ```
  public class NearestNeighboursByFreqUtilityTest
  extends java.lang.Object
  ```

  Class

  Version:
  :   13 May 2012

  Author:
  :   Luna De Ferrari ldf(at)st-andrews.ac.uk or
      luna.deferrari@st-andrews.ac.uk

- - ### Constructor Summary

    Constructors

    Constructor and Description

    `NearestNeighboursByFreqUtilityTest()`
  - ### Method Summary

    Methods

    | Modifier and Type | Method and Description |
    | --- | --- |
    | `static NearestNeighboursByFreqUtility` | `getNNUtilityTestSet()` |
    | `static NearestNeighboursByFreqUtility` | `getNNUtilityTrainSet()` |
    | `void` | `testGetFreqToInstancesMap()` |
    | `void` | `testGetFreqToInstancesMap2()` |
    | `void` | `testGetInstanceToFeatureMap()` |
    | `void` | `testGetInstanceToFeatureMap2()` |
    | `void` | `testGetLinearNNSearch()` Test method for `NearestNeighboursByFreqUtility.getLinearNNSearch()` . |

    - ### Methods inherited from class java.lang.Object

      `equals, getClass, hashCode, notify, notifyAll, toString, wait, wait, wait`

- - ### Constructor Detail


    - #### NearestNeighboursByFreqUtilityTest

      ```
      public NearestNeighboursByFreqUtilityTest()
      ```
  - ### Method Detail


    - #### testGetFreqToInstancesMap

      ```
      public void testGetFreqToInstancesMap()
      ```


    - #### testGetFreqToInstancesMap2

      ```
      public void testGetFreqToInstancesMap2()
      ```


    - #### testGetInstanceToFeatureMap

      ```
      public void testGetInstanceToFeatureMap()
      ```


    - #### testGetInstanceToFeatureMap2

      ```
      public void testGetInstanceToFeatureMap2()
      ```


    - #### testGetLinearNNSearch

      ```
      public void testGetLinearNNSearch()
      ```

      Test method for
      `NearestNeighboursByFreqUtility.getLinearNNSearch()`
      .


    - #### getNNUtilityTestSet

      ```
      public static NearestNeighboursByFreqUtility getNNUtilityTestSet()
      ```


    - #### getNNUtilityTrainSet

      ```
      public static NearestNeighboursByFreqUtility getNNUtilityTrainSet()
      ```


- Overview
- Package
- Class
- Use
- Tree
- Deprecated
- Index
- Help

- Prev Class
- Next Class

- Frames
- No Frames

- All Classes

- Summary:
- Nested |
- Field |
- Constr |
- Method

- Detail:
- Field |
- Constr |
- Method
